# New-onset migraine with aura after transcatheter closure of atrial septal defect

**DOI:** 10.1007/s10194-012-0458-1

**Published:** 2012-05-24

**Authors:** Yuji Kato, Daisuke Furuya, Hirotaka Ishido, Toshiki Kobayashi, Norio Tanahashi

**Affiliations:** 1 Department of Neurology and Cerebrovascular Medicine, Saitama International Medical Center, Saitama Medical University, 1397-1 Yamane, Hidaka, Saitama 350-1298 Japan; 2 Department of Pediatric Cardiology, Saitama International Medical Center, Saitama Medical University, 1397-1 Yamane, Hidaka, Saitama 350-1298 Japan

**Keywords:** Migraine, Atrial septal defect, Transcatheter closure, Amplatzer septal occluder

## Abstract

Transcatheter closure of atrial septal defect (ASD) is associated with a high success rate and become an accepted alternative to surgical treatment. We describe here a case of a 35-year-old woman who presented with migraine attacks with aura after transcatheter closure of ASD with an Amplatzer septal occluder device. We postulate that any of the following may have been responsible for her condition: platelet activation on the surface of the device, nickel allergy, or the release of the atrial natriuretic peptide associated with the stretch of the atrial septum caused by the device. This case demonstrates that de novo migraine can occur after transcatheter closure of ASD and should be recognized as a potential complication.

## Introduction

Several studies have demonstrated a significant reduction or even an abolition of migraine headache attacks (MHAs) after transcatheter closure of atrial septal defect (ASD) or patent foramen ovale (PFO) [1]. In the MIST trial, the only prospectively randomized, double-blind controlled study, no significant reduction in the prevalence of migraine after PFO closure was found, although a significant reduction in headache days was seen in patients with severe, therapy-resistant migraine [2]. In contrast, it has been reported that new-onset MHA or exacerbation of migraine in prior migraine sufferers can also occur after atrial septal device implantation in adult and pediatric patients [3–6]. However, only a few studies systematically investigated the characteristics of new-onset migraine attack occurrence after atrial septal device implantation [6]. We describe here a case of a 35-year-old woman who presented with migraine attacks with aura after transcatheter closure of ASD with an Amplatzer septal occluder device.

## Case report

A 35-year-old female with a negative family history and personal history of headaches or other illness of interest was seen to have abnormal ECG findings (right axis deviation and right ventricular hypertrophy) during the course of a routine medical checkup. She was asymptomatic and was referred to our institution for further cardiac evaluation. Following transthoracic echocardiography, she was diagnosed with an ostium secundum ASD with significant left-to-right shunting (approximate *Q*_p_/*Q*_s_ ratio of 2:1). It was seen to measure 12 mm in stop-flow diameter by transesophageal echocardiography. Transcatheter closure of the defect was performed with an Amplatzer septal occluder device (15 mm). Treatment with aspirin (200 mg) was started 24 h before the procedure. The next morning, the patient began to notice blurry vision in the left eye for 20 min several times a day. Two days later, the patient developed scintillating scotoma, which gradually expanded over 5–20 min, followed by a pulsating headache in the bilateral forehead accompanied by photophobia, which improved spontaneously after ~4 h.

**Fig. 1:**
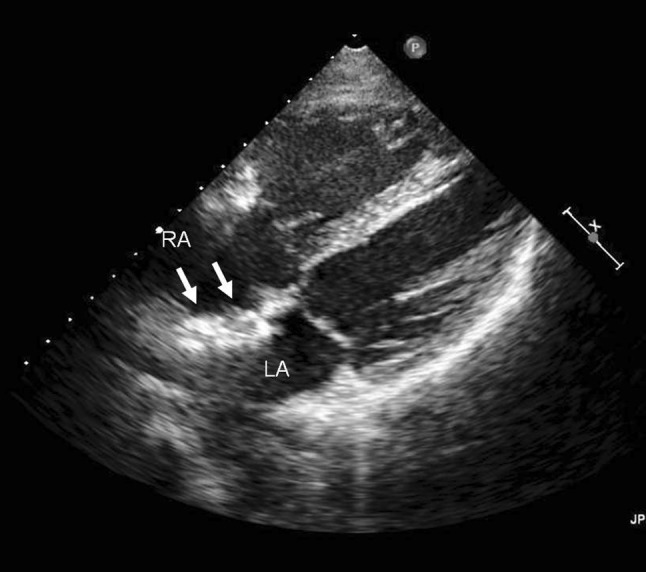
Echocardiogram at the onset of migraine. The device (*arrows*) was positioned in the atrial septum with no evidence of thrombus. *RA* right atrium, *LA* left atrium

Subsequently, she suffered similar headaches twice a week. The intensity and duration of headache increased and were made worse by routine physical activity and nausea. As a result of these symptoms, cerebral magnetic resonance imaging was performed, the result of which was normal. She fulfilled International Headache Society criteria for typical aura with migraine headache (ICHD-II code 1.2.1). Numerous transthoracic echocardiography failed to reveal any images suggestive of thrombus on the device surface or in the cardiac cavities (Fig. 1), though post-operative minor leakage left-to-right shunting through the device was detected. A transthoracic echocardiography performed 2 months after ASD closure did not reveal any residual shunt. The minor leakage may cause platelet activation. Unfortunately, we did not perform transcranial Doppler. We switched from aspirin to sarpogrelate hydrochloride (300 mg/day) and then to clopidogrel (75 mg/day). Although clopidogrel was continued for 3 months, the patient had no response with resolution of her headaches. During some MHA, the patient took eletriptan as soon as she noticed the headache, and the headache was improved within the first hour. Prophylactic treatment with lomerizine, a calcium channel blocker, was initiated 4 weeks after the onset of migraine headache and continued for 6 months. The frequency of headache attacks gradually decreased and disappeared 9 months after the onset. After a 2-year follow-up, she suffered periodic auras without headache for approximately once a month under no medication.

## Discussion

The possibility of ASD being a predictive risk factor for migraine is less evident than in the case of PFO, although the prevalence of migraine in patients with ASD seemed to be increased compared to the general population [7]. Nevertheless, cases of de novo migraine or exacerbated migraine with aura in prior migraine sufferers have been described following percutaneous closure of some ASD [3–6], something that does not appear to hold true in the case of similar treatment procedures for PFO.

De novo development of migraine, mostly with aura, has been reported in single or case series. In a retrospective study using questionnaires, Mortelmans et al. [3] reported an incidence of 19 % of new-onset MHA after transcatheter ASD closure. Similarly, Voet et al. [5] reported an incidence of 12 % that disappeared at long-term follow-up. Sharifi et al. [4] reported response of five post-ASD closure migraineurs to a loading dose of 300 mg clopidogrel. In a retrospective analysis, Rodés-Cabau et al. [6] found de novo migraine in 12 % of patients after ASD closure compared with 0 % after PFO closure. The first migraine episode occurred mostly within 2 weeks after the closing procedure and persisted in 2/3 of the cases for more than 2 years. However, in a prospective study, Riederer et al. [7] did not observe de novo migraine in any patients.

The mechanisms that account for the occurrence of new-onset MHA after ASD closure remain unclear. Platelet activation on the surface of the device has ever been proposed as a mechanism of migraine in these cases. Recently, Nozari et al. [8] demonstrated that microemboli triggered cortical spreading depression without causing microinfarction in a mouse model. Even if there is no brain ischemic defect, microembolic theory cannot necessarily be denied. Also, nickel allergy might be responsible for migraine occurrence or aggravation in these patients [9]. However, neo-endothelialization of an ASD device usually occurs within the first 3 months after device implantation [10], leading to the expectation of the disappearance of migraine attacks in most patients after this period of time, if transient platelet activation and/or nickel release were the triggers for migraine in these cases. As MHAs in the present case persisted for over 3 months and both clopidogrel and sarpogrelate hydrochloride were not effective, our data do not support these hypotheses. As another hypothesis, the stretch of the atrial septum caused by the device itself might induce the release of the atrial natriuretic peptide (ANP) from the atrial myocytes, a vasoactive antagonist of vasopressin that has been proposed as a possible link between ASD closure and migraine aggravation [5, 6].

A possible mechanism, therefore, of de novo migraine attacks after ASD in our patient could be associated with elevated or changed level of ANP as a consequence of intra-atrial pressure imbalance after closure. Further mechanistic studies with long-term ANP measurement are needed to clarify the pathophysiologic basis for the occurrence of migraine attacks after ASD closure. We are planning a prospective study about the influence of ASD closure on migraine.
